# Coincidence of low genetic diversity and increasing population size in wild gaur populations in the Khao Phaeng Ma Non-Hunting Area, Thailand: A challenge for conservation management under human-wildlife conflict

**DOI:** 10.1371/journal.pone.0273731

**Published:** 2022-08-30

**Authors:** Prateep Duengkae, Nattakan Ariyaraphong, Wanlaya Tipkantha, Waleemas Jairak, Sudarath Baicharoen, Dung Ho My Nguyen, Onjira Korboon, Worapong Singchat, Thitipong Panthum, Syed Farhan Ahmad, Erngsiri Kaewkhunjob, Chavin Chaisonkhram, Umaporn Maikaew, Narongrit Muangmai, Gittiyaporn Ieamsaard, Supaphen Sripiboon, Paanwaris Paansri, Warong Suksavate, Aingorn Chaiyes, Supagit Winitpornsawan, Umphornpimon Prayoon, Thiti Sornsa, Ratchanee Chokcharoen, Annop Buanual, Boripat Siriaroonrat, Yongchai Utara, Kornsorn Srikulnath

**Affiliations:** 1 Special Research Unit for Wildlife Genomics (SRUWG), Department of Forest Biology, Faculty of Forestry, Kasetsart University, Bangkok, Thailand; 2 Animal Genomics and Bioresource Research Unit (AGB Research Unit), Faculty of Science, Kasetsart University, Bangkok, Thailand; 3 Laboratory of Animal Cytogenetics and Comparative Genomics (ACCG), Department of Genetics, Faculty of Science, Kasetsart University, Bangkok, Thailand; 4 The Zoological Park Organization of Thailand, Bang Sue, Bangkok, Thailand; 5 Interdisciplinary Graduate Program in Bioscience, Faculty of Science, Kasetsart University, Bangkok, Thailand; 6 The International Undergraduate Program in Bioscience and Technology, Faculty of Science, Kasetsart University, Bangkok, Thailand; 7 Department of Fishery Biology, Faculty of Fisheries, Kasetsart University, Bangkok, Thailand; 8 Department of National Parks, Wildlife and Plant Conservation, Bangkok, Thailand; 9 Department of Large Animal and Wildlife Clinical Sciences, Faculty of Veterinary Medicine, Kasetsart University, Kamphaeng Saen Campus, Nakhon Pathom, Thailand; 10 School of Agriculture and Cooperatives, Sukhothai Thammathirat Open University, Nonthaburi, Thailand; 11 Faculty of Environment and Resource Studies, Mahidol University, Bangkok, Thailand; 12 Amphibian Research Center, Hiroshima University, Kagamiyama, Higashihiroshima, Japan; Central University of Punjab, INDIA

## Abstract

The gaur (*Bos gaurus*) is found throughout mainland South and Southeast Asia but is listed as an endangered species in Thailand with a decreasing population size and a reduction in suitable habitat. While gaur have shown a population recovery from 35 to 300 individuals within 30 years in the Khao Phaeng Ma (KPM) Non-Hunting Area, this has caused conflict with villagers along the border of the protected area. At the same time, the ecotourism potential of watching gaurs has boosted the local economy. In this study, 13 mitochondrial displacement-loop sequence samples taken from gaur with GPS collars were analyzed. Three haplotypes identified in the population were defined by only two parsimony informative sites (from 9 mutational steps of nucleotide difference). One haplotype was shared among eleven individuals located in different subpopulations/herds, suggesting very low genetic diversity with few maternal lineages in the founder population. Based on the current small number of sequences, neutrality and demographic expansion test results also showed that the population was likely to contract in the near future. These findings provide insight into the genetic diversity and demography of the wild gaur population in the KPM protected area that can inform long-term sustainable management action plans.

## Introduction

Gaur (*Bos gaurus* Smith, 1827) also known as “Indian bison” or “seladang” is the largest living wild cattle species [1]. Historically, gaur were the main prey of large carnivores and played important roles in maintaining the ecosystem by preventing vegetation overgrowth [2–5]. Gaur once ranged widely throughout mainland South and Southeast Asia, and in 2016, the global population was estimated at 15,000 to 35,000, with mature individuals numbering between 6,000 and 21,000 [6]. During the past century, the wild gaur population has declined by more than 80% due to the loss of suitable habitat to agriculture and poaching for horn and meat. Hybridization between wild gaur and domestic cattle has also resulted in the transmission and outbreak of various diseases, such as foot-and-mouth, rinderpest, and anthrax [6, 7]. Currently, the gaur is listed as globally vulnerable by the International Union for Conservation of Nature and Natural Resources [6]. In Thailand, the gaur was reassigned as an endangered species from a vulnerable species in 2005, and is also a protected wild animal listed in the Wild Animal Reservation and Protection Act (2019) [8, 9]. With the expansion of agricultural areas, settlements, and roads, many wildlife habitats have become fragmented, resulting in small gaur populations in many protected areas [10]. Consequently, gaurs are fast disappearing from northern and southern areas of Thailand [11], and urgent conservation management is required to provide a concrete action plan.

Gaurs are now located in 46 protected areas in Thailand with the highest abundance in the Eastern Forest Complex followed by the Dong Phayayen-Khao Yai, Khlong Sang-Khao Sok, and Western Forest Complexes [11, 12]. Gaurs can also be found in the Dong Phayayen-Khao Yai Forest Complex where the land area supports viable populations, with a high and medium abundance of animal tracks and signs [13]. Several gaur populations inhabit the land between protected areas and surrounding agricultural areas, such as the Khao Phaeng Ma (KPM) Non-Hunting Area and Khao Yai National Park [14, 15]. Around 1990, only 35 gaurs were observed in the KPM, with 96 individuals in 2006, 160 individuals in 2011, 271 individuals in 2016, and 250–300 individuals recorded in 2022 [15–17].

Gaur population recovery has caused conflict with villagers along the border of the KPM protected area as it has generally occurred along forest edges next to farmland. Furthermore, gaurs have a large home range, which may negatively influence broader economic and political aspects of biodiversity conservation [18, 19]. Farmland near forest edges has a high risk of crop damage from gaur populations, and farmers in conflict with wildlife sometimes resort to killing or poisoning, although ecotourism from watching wild gaurs has recently increased [20, 21]. Moreover, outbreaks of foot-and-mouth and lumpy skin disease, which affect both domestic and wild cattle, have also impacted gaurs in Thailand [22].

Information about the current genetic status of the wild gaur population is important and necessary to develop strategies for conservation and effective long-term management in the KPM protected area. Within this context, the principal aims of this study were to (1) measure the genetic diversity in the extant population of wild gaurs in the KPM, and (2) detect the signatures of past and present demographic events in the wild gaur population and predict their future course. The mitochondrial displacement-loop (mt D-loop) region offers a powerful genetic marker that is useful for investigating the origin, genetic diversity, and relationships among cattle populations and species [23–26]. Here, we sampled 13 wild gaurs ranging in the KPM Non-Hunting Area, and their genetic profiles were investigated using mt D-loop sequencing. We also report individual movement data based on microchip implantation of all the examined individuals. Overall, our results provide important information that can inform the maintenance and improvement of future gaur management strategies and conservation planning for gaurs in Thailand.

## Materials and methods

### Landscape information, gaur capture, and microchip implantation

We captured 13 gaurs from the main population in the KPM protected area (14°21’55"N, 101°47’38"E), an 8 km^2^ (2.83 x 2.83 km) area of restored montane forest bordering Khao Yai National Park and part of the Dong Phayayen-Khao Yai Forest Complex [27]. Detailed information on the sampled population is presented in S1 Table. The population in the KPM was recorded as 243–258 individuals between 2020 and 2022 by observations of officers from the Department of National Parks, Wildlife and Plant Conservation (DNP) [17]. The gaur population in KPM is separated into six subpopulation groups, with four mainly dwelling in the KPM and moving between the KPM, Khao Yai National Park, and the surrounding agricultural areas for feeding, while two small subpopulations inhabit fragmented forest patches outside the protected areas [17]. The landscape within 10 km^2^ of the gaur populations has well-developed infrastructure characterized by an agricultural matrix and lacks noteworthy patches of natural vegetation. Land use includes farming of corn, cassava, and other crops; orchards and gardens; plantations; fallows; and various animal farms. The climate in the area is tropical-monsoonal, with a dry season from November to March followed by a hot inter-monsoonal period until May, and a wet season from May to October.

The gaurs were captured by darting from a vehicle or tree platform, and capture and handling were overseen by qualified veterinarians. Satellite collars (VERTEX Lite-5D IRIDIUM Collar, Vectronic Aerospace GmbH, Germany) were fitted to 13 adult gaurs (nine males and four females), and data were collected from both sexes and the different subpopulations [5, 28–31]. All tracked individuals were subjected to general anesthesia to reduce stress and facilitate handling using a combination of thiafentanil oxalate and medetomidine HCl following the modified protocol described by Napier et al. [32]. The GPS tracker serial numbers were used as the gaur identifications (IDs). While under anesthesia, the animals were closely observed and monitored for vital signs under the supervision of the veterinarian team. To investigate herd grouping within the population, all animals were implanted with a subcutaneous microchip in the neck area. Blood specimens were also collected from the coccygeal or jugular vein using a Vacuette® 18-gauge needle containing 5 ml EDTA (pH 8.0, 1.2–2.0 mg EDTA/1 ml of blood) (Greiner Bio-One, Kremsmünster, Austria) for DNA extraction. After all the procedures were completed, the animals were treated with reversal drugs and remotely observed until full recovery. All animal care and experimental procedures were approved by the Animal Experiment Committee, Zoological Park Organization (ZPO) (Approval no. 78109) and Kasetsart University (Approval no. ACKU65-SCI-08) and conducted in accordance with the Regulations on Animal Experiments at ZPO and Kasetsart University. Permission to tag the animals was granted by the Department of National Parks, Wildlife and Plant Conservation, Ministry of Natural Resources and Environment, Thailand (DNP 0907.4/11255).

### Mitochondrial D-loop sequencing

Whole genomic DNA was extracted following the standard phenol-chloroform protocol with slight modifications for different tissues, as previously described Srikulnath et al. [33]. The DNA quality and concentration were determined using a spectrophotometer (NanoDrop™ 2000, Thermo Scientific, Wilmington, DE, USA). The mt D-loop fragments were amplified following the method of Kathiravan et al. [34] using the primers Mito (D-loop) F (5′–TAGTGCTAATACCAACGGCC–3′) and Mito (D-loop) R (5′–AGGCATTTTCAGTGCCTTGC–3′). Each PCR amplification was performed using 15 μl of 1× ThermoPol® buffer containing 1.5 mM MgCl_2_, 0.2 mM dNTPs, 5.0 μM primers, 0.5 U Taq polymerase (Apsalagen Co., Ltd, Bangkok, Thailand), and 25 ng genomic DNA. The PCR conditions were as follows: initial denaturation at 94°C for 3 min, followed by 35 cycles of 94°C for 30 s, 55°C for 30 s, 72°C for 30 s, and a final extension at 72°C for 5 min. The PCR products were detected by electrophoresis through 1% agarose gel. The DNA fragments were extracted from the ethidium bromide stained gel and purified using a QIAquick Gel Extraction Kit (QIAGEN GmbH, Hilden, Germany). Nucleotide sequences of the DNA fragments were determined by the DNA sequencing service of First Base Laboratories Sdn Bhd (Seri Kembangan, Selangor, Malaysia). The blastn and blastx programs (http://blast.ncbi.nlm.nih.gov/Blast.cgi) were used to search nucleotide sequences in the National Center for Biotechnology Information database (nr) to confirm the identity of the amplified DNA fragments. The sequences generated were deposited in the DNA Data Bank of Japan (DDBJ) (https://www.ddbj.nig.ac.jp/) (Accession numbers: LC707734 –LC707746) (S2 Table).

### Sequence analysis

Multiple sequence alignment was performed for 19 sequences in the mt D-loop dataset including nine males, four females, and six *B*. *gaurus* sequences retrieved from GenBank (GenBank accession numbers: MG018948, MN365659, HM215246, AF083371, MK584901, and MK584900). The sequences were aligned using the “Clustal” default parameters of the Molecular Evolutionary Genetics Analysis 11 (MEGA11) software [35]. All unalignable and gap-containing sites were carefully removed and trimmed from the datasets. Estimates of haplotype (*h*) diversity and nucleotide (π) diversity [36], the number of haplotypes (*H*), the estimator theta (*S*), the overall haplotype, and the average number of nucleotide differences (*k*) were calculated based on the mt D-loop sequences, as implemented in DnaSP version 6 [37]. A statistical parsimony network of the consensus sequences was constructed using the Templeton, Crandall, and Sing (TCS) algorithm implemented in PopART version 1.7 to examine haplotype grouping and population dynamics [38].

Demographic history was examined using statistical tests of neutrality as Tajima’s *D* [39], Fu and Li’s D* and F* tests [40], Fu’s *F*_*s*_ [41], Ewens-Watterson test and Chakraborty’s test, and calculated using Arlequin version 3.5.2.2 [42]. Ramos-Onsins and Rozas’s *R*_*2*_, which has greater statistical power for small sample sizes, was calculated using DnaSP version 6 [43]. The significance of the differences among these values was determined using 10,000 coalescent simulations in accordance with the recommended software parameters. To test for genetic signatures of historical population expansion within the wild gaur population, we used the mismatch distribution approach, in which an observed frequency distribution of pairwise nucleotide differences was obtained among individuals with expected distributions from an expanding population (small raggedness index) or a stationary population (large raggedness index) [44, 45]. These models were applied to estimate population expansion parameters using a generalized least-squares approach and to compute confidence intervals by bootstrapping (10,000 replicates) implemented in DnaSP version 6. Bayesian coalescent-based methods were then performed to evaluate the historical demographic fluctuations using the Extended Bayesian Skyline Plot (EBSP) method implemented in BEAUTi version 2.0.2 (part of the BEAST version 2.0.2 package) [46, 47]. This involved applying the HKY model, strict clock, and Coalescent Bayesian Skyline Model with a Gamma-distribution prior. For the mean substitution rate, the prior was set as a lognormal distribution with a mean of 0.626% per million years and a standard deviation of 0.516% per million years to match the rate estimated from fossil data [48, 49]. TRACER was applied to assess burn-in and the effective sample sizes (ESS) of the parameters [50]. The EBSP method allowed us to fit different demographic scenarios by allowing changes in population size over time.

### Forward genetic simulation

To simulate guar population genetic scenarios, individual-based forward genetic simulations were performed using the simulation program quantiNEMO an individual-based sequence data input [51]. The simulation estimated future genetic variation and diversity, thereby implying genetic fitness [52]. In this study, four scenarios with different carrying capacities were set at a 50% decrease (125), fixed at the current population size (250), a 50% increase (375), and a 100% increase (500). These scenarios represent future management practices dealing with conflict between humans and gaur in the area by either controlling the population or via mutualistic scenarios. Each simulation was run for 300 generations with 1,000 replicates.

## Results

### Status of the wild gaur population in the KPM protected area

**Fig 1:**
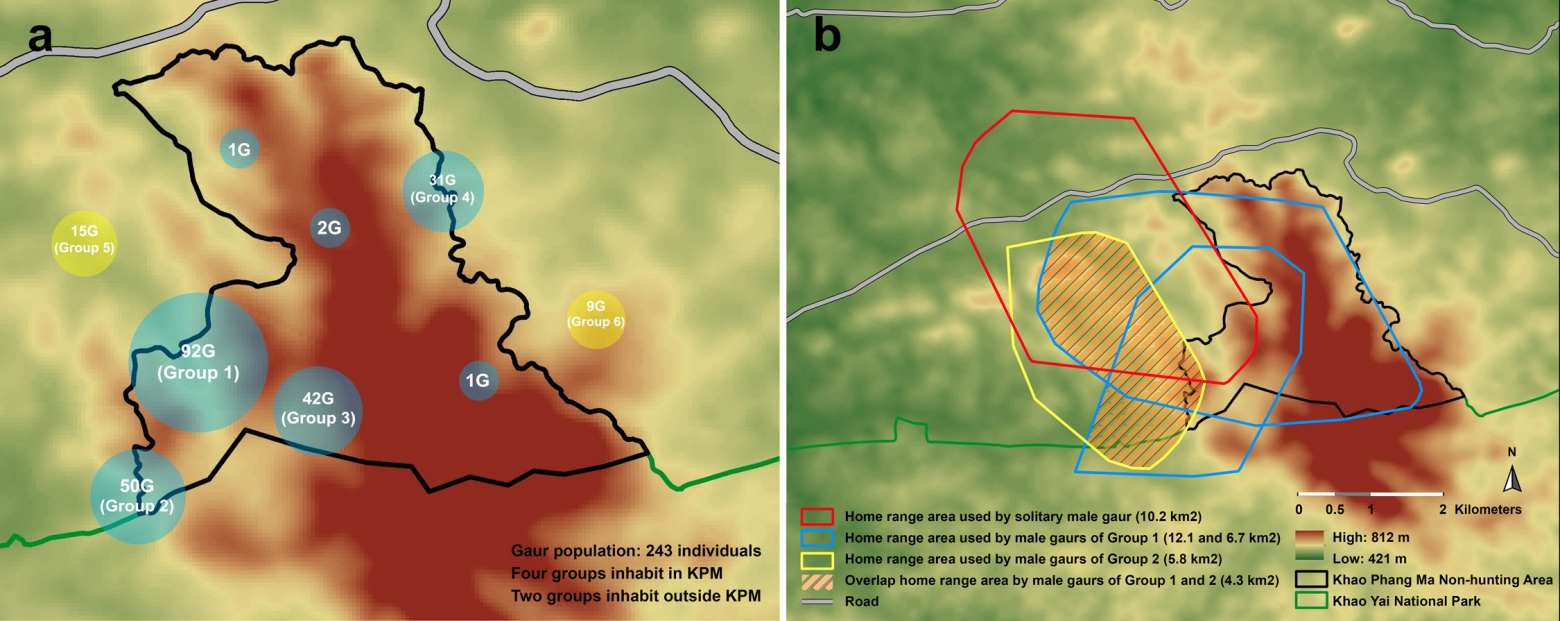
Global positioning system (GPS) tracks. Thirteen wild gaurs in Khao Phaeng Ma (KPM) Non-Hunting Area, Thailand, 2021. (a) The gaur population group in KPM is divided into six subpopulations (Groups 1–6): a blue circle represents the groups dwelling in KPM, and a yellow circle represents the groups living outside the protected area; “xxxG” indicates the number of gaurs in each herd, (b) The overall home range area of male gaurs (Group 1 and Group 2), and their area of overlap in protected areas and agricultural areas.

Based on observation by DNP officers, the original herd (Group 1) inhabiting the KPM protected area has separated into four herds (Groups 1–4) within the protected area and two satellite herds (Groups 5 and 6) living outside KPM as well as two solitary adult males (Fig 1). Thirteen adult gaurs were captured and deployed with GPS collars. Based on GPS data, the individuals moved between the protected areas and the surrounding agricultural land, concurring with visual observations. Most of the wild gaurs ranged within an agricultural landscape area of 8–10 km^2^.

### Genetic diversity and demography of the wild gaur populations

**Fig 2:**
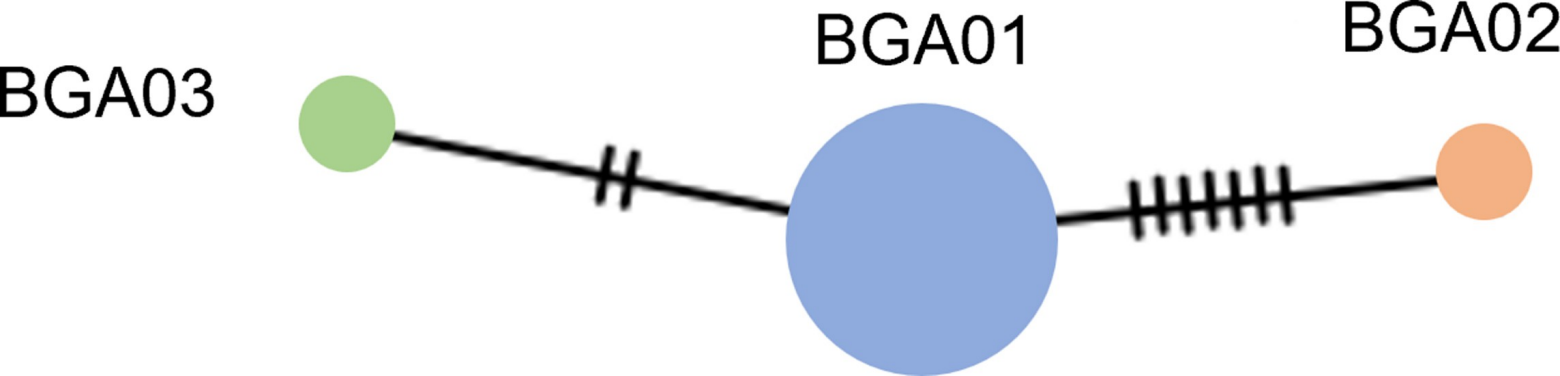
Haplotype network derived from nucleotide data for mitochondrial D-loop sequencing of wild bull (*Bos gaurus*, Smith, 1827) for 13 individuals. Different colors distinguish the samples. Each circle represents a unique DNA sequence (haplotype), with the circle diameter reflecting the total number of individuals possessing the haplotype.

**Fig 3:**
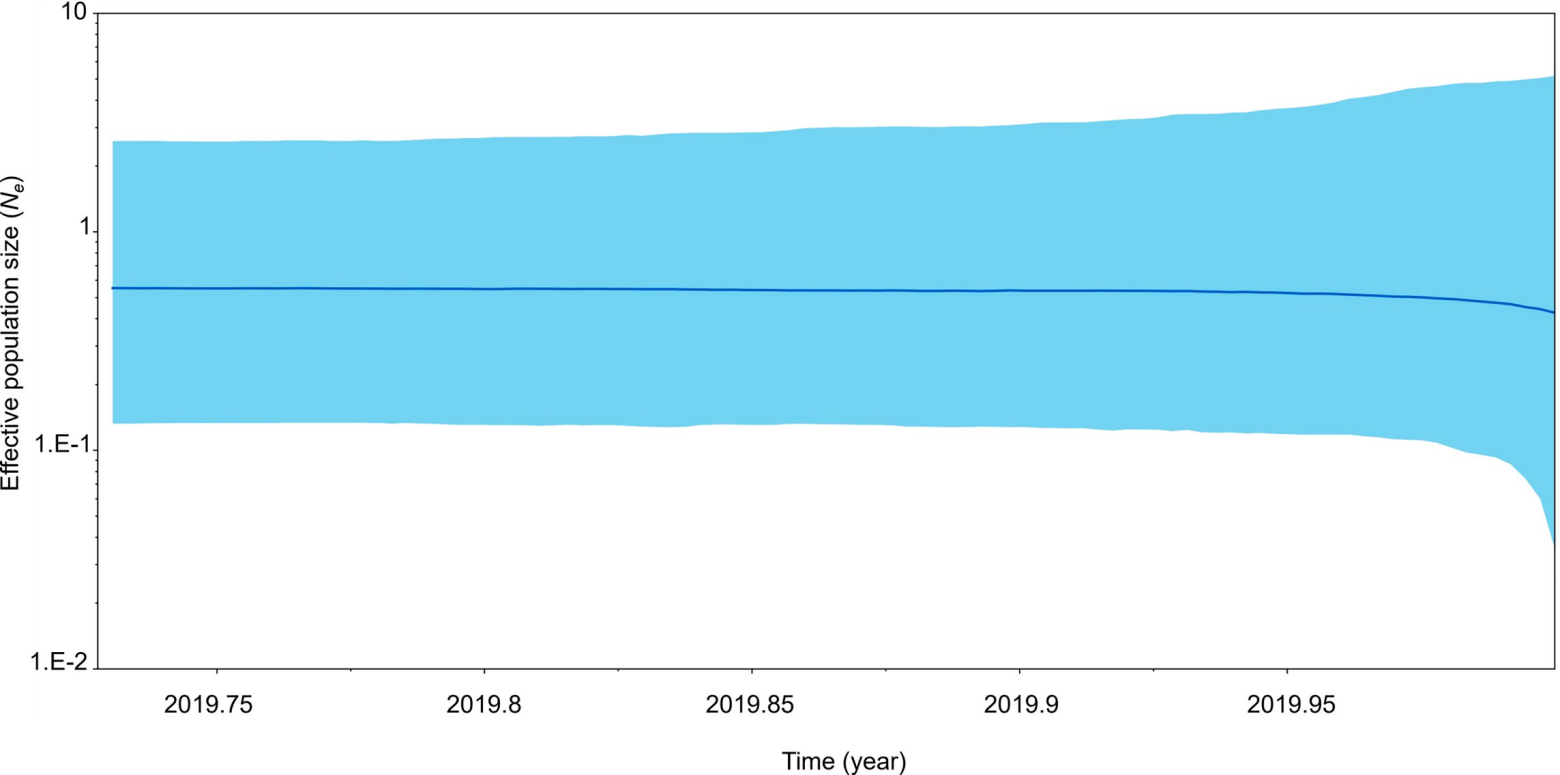
Coalescent Bayesian Skyline analysis output. The black line represents the median estimated effective population size, while blue areas represent the upper and lower bounds of the 95% higher posterior density interval. The x-axis is time and the y-axis is a log scale.

The amplicon length and alignment length of the mt D-loop sequences were 1,200 and 1,054 bps, with overall haplotype and nucleotide diversities of 0.295 ± 0.157 and 0.006 ± 0.003, respectively. A simple haplotype network was constructed from the large number of detected polymorphic sites and haplotypes. Three haplotypes were observed from the mt D-loop sequences, and the most common was BGA01, with 11 individuals (Fig 2). Five different tests of neutrality were used to examine historical population reduction and expansion of the wild population: the Tajima’s *D* value was -2.134, *p* < 0.01; the Fu’s *FS* value was 7.165, *p* = 0.993; the Fu and Li’s F* value was -2.814, *p* < 0.05; the Fu and Li’s D* value was -2.615, *p* < 0.05; the Ewens-Watterson value was 1.000, *p* = 1.000; and the Ramos-Onsins and Rozas’s *R*_*2*_ value was 0.211. Mismatch distribution analysis indicated a multimodal distribution (S1 Fig), while the population had a raggedness index (0.577, *p* = 0.900). The EBSPs suggested that population size remained constant over a long period (Fig 3).

### Demographic future simulation

**Fig 4:**
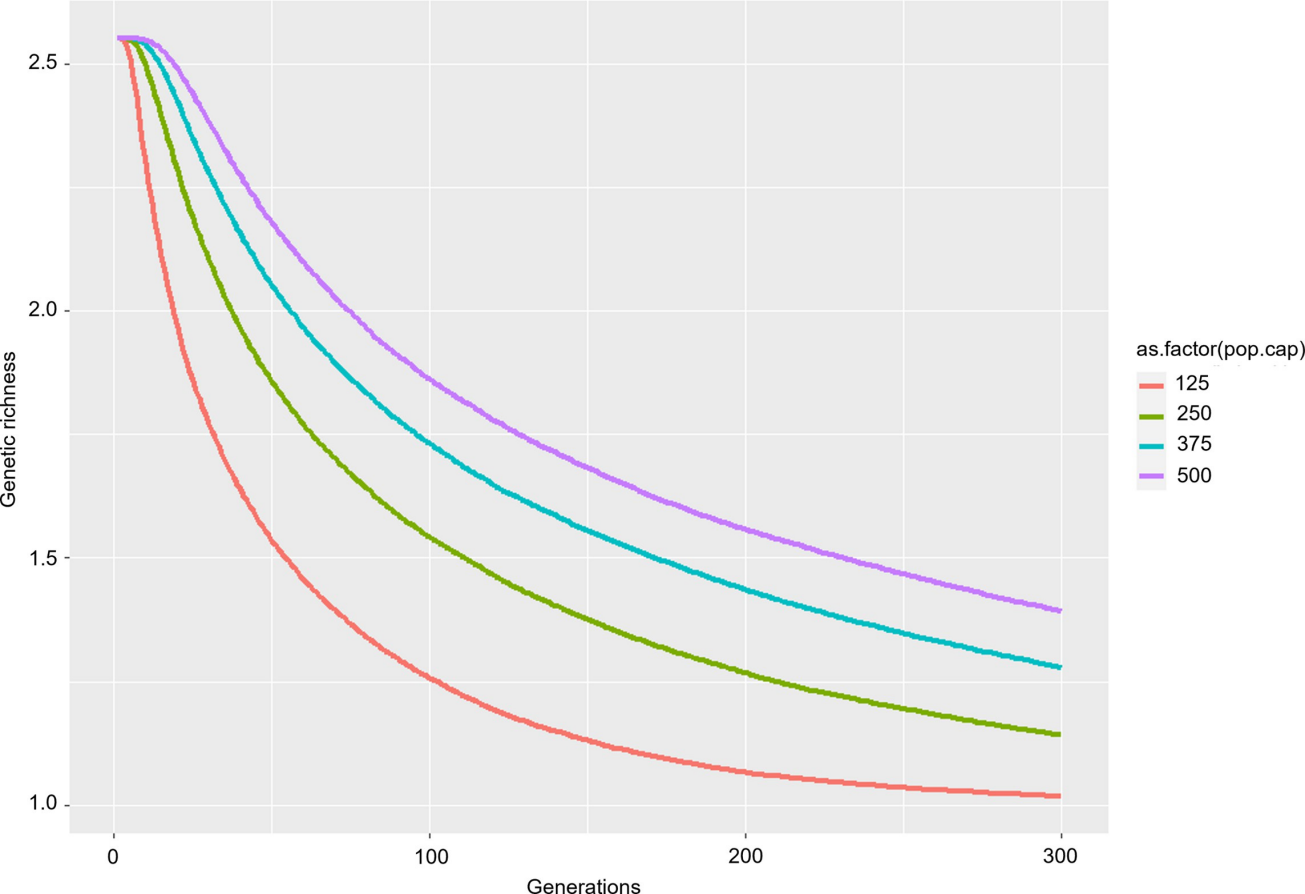
Simulation results showing the relationship between generations and genetic diversity.

Forward genetic simulations were performed for four scenarios of varied carrying capacity of gaur populations in the KPM Non-Hunting Area (Fig 4). The results from 100 simulated generations showed a decrease in genetic diversity, which became fixed by approximately 300 generations. Genetic diversity declines were slower when wild populations have a higher carrying capacity, and faster with lower carrying capacity.

## Discussion

Wildlife is facing multiple extinction threats, mainly because of global climate change and increasing anthropogenic activities associated with habitat loss [53–57]. This is very serious in the context of global conservation biology, which requires blueprints for achieving a better and more sustainable future for all following the UN Sustainable Development Goals (SDGs); SDG 13 (“Take urgent action to combat climate change and its impacts”), SDG 15 (“Protect, restore and promote sustainable use of terrestrial ecosystems, sustainably manage forests, combat desertification, and halt and reverse land degradation and halt biodiversity loss”) and SDG 17 (“Strengthen the means of implementation and revitalize the Global Partnership for Sustainable Development”) are all relevant in this context. Wild gaur play a crucial ecological role in dry deciduous forests by maintaining physical habitat structure and were once a key component of the food chain in tiger-occupied landscapes [58]. An increase in the wild gaur population in the KPM protected area has been well documented as an example of population recovery causing conflict with villagers along the borders of the non-hunting area. Here, we studied the genetic diversity in this population to inform wild gaur management. The mt D-loop sequences of 13 gaurs revealed low nucleotide diversity despite high haplotype diversity. Six gaurs shared haplotype BGA01, including the two solitary adult males, while only three haplotypes (with a 2 bp of parsimonious informative sites) were observed from a total of 1,054 bp, carrying a value of 0.19%. This variation was lower than that found in Malayan gaur, with a variation of 13.25% within the population [59], suggesting very few maternal lineages in the KPM population. A lack of mitochondrial DNA variation in the wild gaur population suggests a small population with a low number of founder females in the KPM. However, only two parsimony informative characters were detected among our three sequence variations. This suggests that inbreeding has occurred, which is unsurprising considering a founder population of just 35 individuals in 1990 and a current population of 250–300.

In our demographic analyses of three out, five different tests of neutrality showed statistical significance, while the nonsignificant raggedness index indicated recent bottlenecks and a sudden recent population expansion. By contrast, the mismatch distribution analysis indicated a multimodal distribution, suggesting demographic equilibrium or population stability, as also revealed by the Bayesian Skyline plots. These results suggest a stable wild population showing a decreasing trend rather than the results predicted by the mismatch distribution. Considering the historical records, the wild gaur population in the KPM Non-Hunting Area has likely experienced both bottleneck and expansion events during the last 30 years of population recovery. The population has become relatively constant over the last several years, and our results suggest a tendency toward contraction in the near future. It is likely that a decrease in genetic diversity became fixed in this population over the course of 300 generations.

The gaur is a gregarious animal, with a social herd organization including sub-adult males, adult females, juveniles, and calves [11]. Adult females have maximum influence on group size as the matriarchs, and adult males can be solitary. The KPM protected area consists of reforested montane habitat covering 8 km² [27], providing a range of environmental goods and services vital to economic ecotourism development both at the ‘ecological scale’ and at the ‘community level’ [60]. The KPM has the potential for a variety of positive environmental, socio-cultural, and economic impacts that can provide mutual benefits to conservation, tourism, and local people. This offers a potential catalyst to bring positive change and development at both local and national levels [60]. The KPM protected area is the best place to view wild gaurs in their natural grassland habitats in Thailand; however, our results indicate the occurrence of low genetic diversity in this population, with few maternal lineages and a high tendency for population contraction. Space limitations in the KPM and the surrounding agricultural areas are also a concern, as exemplified by the effect of habitat fragmentation on the genetic diversity and differentiation in spatially separated tiger populations in Central India [61, 62]. Human-induced disturbance, including the conversion of wild habitat into agricultural land by native tribes, is also responsible for severe habitat destruction in Central Thailand. Indeed, we suggest that the low mitochondrial diversity and potential population contraction of the contemporary wild gaur population in the KPM are probably the outcomes of extreme habitat loss. This leads to concern that the main wild gaur population is tending toward extinction in the region. Such an outcome would be devastating, destroying local economies built on ecotourism alongside the loss of biodiversity and ecological balance.

Urgent conservation management interventions are necessary to improve the genetic diversity of the KPM gaur population, including translocations from other populations. Genetic monitoring of other populations is also necessary to identify their genetic resources. Increasing the carrying capacity of the population would also help maintain genetic diversity. Strengthening the management of wildlife corridors for gaur and other endangered species in forest complexes is another important measure that can aid animal conservation [27]. With appropriate action, the effective management of gaurs and their habitat might result in the KPM becoming a stronghold for this species. To assist this aim, the population status, carrying capacity, and movement of gaur in the KPM protected area should be further studied to assess habitat potential. Group formation and the age structure of a population would also be useful keys to unlocking the dynamics of population growth and estimating life-history parameters [63–65]. The age structure of a population can be expressed as an interrelated aspect of the distribution of individuals reflecting fecundity, mortality, reproductive status, and population change. Moreover, management plans for suitable areas outside the KPM protected area are also necessary to further develop the protection status of the gaur populations. Currently, an outbreak of foot-and-mouth and lumpy skin diseases is affecting both domestic and wild cattle in Thailand [22]. Active surveillance and remedial action should, therefore, be implemented in suitable areas of gaur habitat as soon as possible.

## Conclusions

Our study highlights some key points regarding the genetic diversity of the guar population in the KPM protected area and recommends several conservation and monitoring measures. Very low genetic diversity with a tendency for population contraction may further negatively impact this wild gaur population, which is vulnerable to extinction. Therefore, conservation management should avoid low genetic distance among individuals wherever possible, while the addition of other wild gaur populations will increase genetic diversity. Increasing the area of suitable habitat will also improve the probability of species survival. Overall, our research provides important new understanding that can inform wild gaur conservation efforts, offering data that can guide those authorities responsible for the conservation planning of wild gaur populations in the KPM protected area and elsewhere. Most importantly, our results emphasize that action must be taken now to ensure a sustainable future for the wild gaur population in the region.

## Supporting information

S1 Table Detailed information of the sampled gaur (*Bos gaurus*, Smith, 1827). (DOCX)

S2 Table Specimen populations of gaur (*Bos gaurus*, Smith, 1827) in the Khao Phaeng Ma (KPM) Non-Hunting Area. All sequences were deposited in the DNA Data Bank of Japan (DDBJ).
(DOCX)

S1 Fig Mismatch distributions analysis. The x-axis represents the number of pairwise differences (mismatches), while the y-axis represents the frequency of these differences. The observed mismatch distribution (red line) is compared to the expected distribution (blue line) for a stable population.
(TIFF)
