# Supplementary material for: Coincidence of low genetic diversity and increasing population size in wild gaur populations in the Khao Phaeng Ma Non-Hunting Area, Thailand: A challenge for conservation management under human-wildlife conflict
Source: PLoS One. 2022 Aug 30;17(8):e0273731. doi: 10.1371/journal.pone.0273731 (PMC9426942; doi:10.1371/journal.pone.0273731)
Supplement: S1 Table — (DOCX) [file pone.0273731.s001.docx]

**S1 Table.** Detailed information of the gaur (*Bos gaurus*, Smith, 1827).

| Collar ID No. | House name | Capture date | Sex | Length (mm) | | | | | Group |
| --- | --- | --- | --- | --- | --- | --- | --- | --- | --- |
|  |  |  |  | Head and  body | Tail | Ear | Neck | Shoulder  height |  |
| 35351 | Sai Fon | 28-Sep-20 | Female | 2,820 | 680 | 240 | 1,020 | - | Group 1 |
| 35352 | Sai Mok | 29-Sep-20 | Male | 2,900 | 910 | 300 | 1,165 | - | Group 1 |
| 35346 | Sai Fha | 08-Apr-21 | Male | 2,900 | 680 | 230 | 1,060 | 900 | Group 1 |
| 35348 | Thong Dee | 09-Apr-21 | Male | 3,530 | 680 | 240 | 1,480 | - | Solitary |
| 35350 | Sai Nam | 15-Jun-21 | Male | 3,400 | 800 | 220 | 1,230 | - | Group 1 |
| 35352 | Sai Rung | 16-Jun-21 | Female | 2,900 | 650 | 220 | 900 | - | Group 2 |
| 35353 | Sai Deed | 19-Sep-21 | Male | - | - | - | 1,400 | - | Group 2 |
| 35349 | Sai Lui | 20-Sep-21 | Male | 2,900 | 730 | 220 | 1,250 | - | Group 2 |
| 35354 | Sai Nao | 21-Dec-21 | Male | 2,150 | 680 | 210 | 1,150 | 1,070 | Group 1 |
| 35355 | Sai Chill | 21-Dec-21 | Male | 2,700 | 880 | 210 | 1,000 | 1,050 | Group 1 |
| 3685 | Sai Tarn | 21-Sep-21 | Female | 2,800 | 720 | 230 | 1,040 | - | Group 1 |
| 3686 | Sai Mai | 22-Dec-21 | Female | 2,580 | 610 | 230 | 900 | 1,400 | Group 1 |
| 3684 | Sai Yud | 22-Dec-21 | Male | - | - | - | 1,250 | - | Group 1 |
