# Supplementary material for: Coincidence of low genetic diversity and increasing population size in wild gaur populations in the Khao Phaeng Ma Non-Hunting Area, Thailand: A challenge for conservation management under human-wildlife conflict
Source: PLoS One. 2022 Aug 30;17(8):e0273731. doi: 10.1371/journal.pone.0273731 (PMC9426942; doi:10.1371/journal.pone.0273731)
Supplement: S2 Table — All sequences were deposited in the DNA Data Bank of Japan (DDBJ). (DOCX) [file pone.0273731.s002.docx]

**S2 Table.** Specimen populations of guar (*Bos gaurus*, Smith, 1827) in the Khao Phang Ma (KPM) Non-hunting Area. All sequences were deposited in the DNA Data Bank of Japan (DDBJ).

| No. | Abbreviation/  Code | Specimen |  | Mitochondrial D-loop DDBJ accession number |
| --- | --- | --- | --- | --- |
|  |  |  | Sex |  |
| 1 | BGA01 | Sai Fha | Male | LC707734 |
| 2 | BGA02 | Sai Deed | Male | LC707735 |
| 3 | BGA03 | Sai Fon | Female | LC707736 |
| 4 | BGA04 | Sai Nam | Male | LC707737 |
| 5 | BGA05 | Sai Rung | Female | LC707738 |
| 6 | BGA06 | Thong Dee | Male | LC707739 |
| 7 | BGA07 | Sai Lui | Male | LC707740 |
| 8 | BGA08 | Sai Tarn | Female | LC707741 |
| 9 | BGA09 | Sai Mok | Male | LC707742 |
| 10 | BGA10 | Sai Yud | Male | LC707743 |
| 11 | BGA11 | Sai Chill | Male | LC707744 |
| 12 | BGA12 | Sai Mai | Female | LC707745 |
| 13 | BGA13 | Sai Nao | Male | LC707746 |
