# Supplementary figures and images for: Coincidence of low genetic diversity and increasing population size in wild gaur populations in the Khao Phaeng Ma Non-Hunting Area, Thailand: A challenge for conservation management under human-wildlife conflict
Source: PLoS One. 2022 Aug 30;17(8):e0273731. doi: 10.1371/journal.pone.0273731 (PMC9426942; doi:10.1371/journal.pone.0273731)

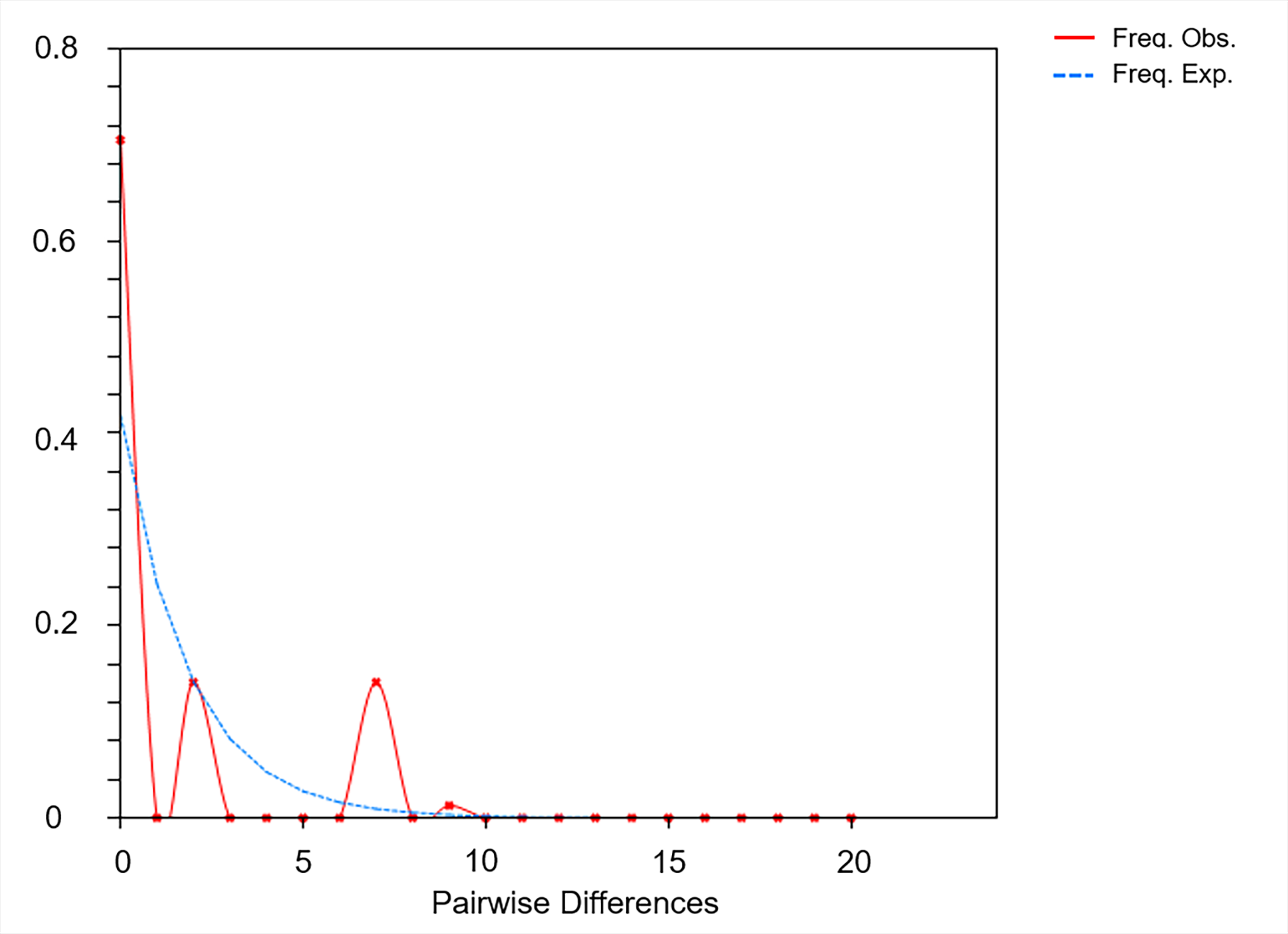

Supplement: S1 Fig — The x-axis represents the number of pairwise differences (mismatches), while the y-axis represents the frequency of these differences. The observed mismatch distribution (red line) is compared to the expected distribution (blue line) for a stable population. (TIFF) [file pone.0273731.s003.tiff]
